# A fluorescence polarization assay for high-throughput screening of inhibitors against HIV-1 Nef-mediated MHC-I downregulation

**DOI:** 10.1016/j.jbc.2024.107529

**Published:** 2024-07-01

**Authors:** Mohammad Karimian Shamsabadi, Xiaofei Jia

**Affiliations:** 1 Department of Chemistry and Biochemistry, University of Massachusetts Dartmouth, Dartmouth, Massachusetts, USA; 2 The Biomedical Engineering and Biotechnology Program, University of Massachusetts Dartmouth, Dartmouth, Massachusetts, USA

**Keywords:** HIV, Nef, MHC-I, antiretroviral, high-throughput screening, fluorescence polarization

## Abstract

The multifunctional, HIV-1 accessory protein Nef enables infected cells to evade host immunity and thus plays a key role in viral pathogenesis. One prominent function of Nef is the downregulation of major histocompatibility complex class I (MHC-I), which disrupts antigen presentation and thereby allows the infected cells to evade immune surveillance by the cytotoxic T cells. Therapeutic inhibition of this Nef function is a promising direction of antiretroviral drug discovery as it may revitalize cytotoxic T cells to identify, and potentially clear, hidden HIV-1 infections. Guided by the crystal structure of the protein complex formed between Nef, MHC-I, and the hijacked clathrin adaptor protein complex 1, we have developed a fluorescence polarization-based assay for inhibitor screening against Nef’s activity on MHC-I. The optimized assay has a good signal-to-noise ratio, substantial tolerance of dimethylsulfoxide, and excellent ability to detect competitive inhibition, indicating that it is suitable for high-throughput screening.

HIV-1 infections, if untreated, lead to acquired immunodeficiency syndrome (AIDS), which is marked by damaged immune system, increased chance of opportunistic infections, other severe illnesses, and death of the patients typically within 3 years. Although antiretroviral therapy (ART) has transformed the treatment of HIV infections and significantly reduced the death rate, currently available antiretrovirals cannot cure the infection. Latent viral reservoirs persist in the infected individuals and can lead to viral rebounds if ART is stopped (1). The life-long use of antiretrovirals, however, often leads to drug resistance and/or severe side effects (2). Novel antiretrovirals that can better treat, and ideally cure, HIV infections are therefore highly desired.

The HIV-1 accessory protein Nef is a small, multifunctional protein that plays an important role in viral pathogenesis (3). Nef expression *in vivo* is associated with high viral loads and disease progression into AIDS (4). Individuals infected by *nef*-defective HIV-1 strains do not develop AIDS for decades in the absence of ART (5, 6, 7, 8). This effect on viral pathogenesis presumably comes from Nef’s ability to enable immune evasion. Nef downregulates major histocompatibility complex class I (MHC-I) from the cell surface, which disrupts antigen presentation and thus enables the infected cell to hide from the immune surveillance mediated by the CD8^+^ cytotoxic T lymphocytes (9, 10). Nef also downregulates the CD4 receptor from the cell surface (11, 12, 13), which facilitates viral replication (14, 15, 16, 17) and, importantly, allows the infected cell to evade antibody-dependent cellular cytotoxicity (18, 19). The promise of Nef inhibition, therefore, is that it should revitalize these essential immune mechanisms to target and kill infected cells. Nef inhibition may be particularly relevant, or even necessary, in the *shock and kill* strategy (20). As proposed, the first step of this strategy is latency reversal, in which viral replication is reactivated therapeutically in infected cells (*shock*). Subsequently, host immunity would detect the cells that are undergoing active HIV-1 replication and clear them (*kill*). It is conceivable that, without inhibiting Nef, the “*kill*” step would be inefficient due to Nef-mediated immune evasion.

The benefits of Nef inhibition may also go beyond the scope of “*kill”*; it may facilitate latency reversal as well. It is known that Nef expression persists during ART (21, 22). A recent study showed that, during the early stage of ART, the immune evasion activity of Nef—particularly the activity to cause MHC-I downregulation and thus evasion of cytotoxic T lymphocytes—correlates positively with viral reservoir size (23). Furthermore, in another study where observations were made after long-term ART, Nef-mediated immune evasion was found to protect genetically intact proviruses and thus contribute to HIV-1 persistence in effector memory CD4+ T cells (24). These findings suggest that successful inhibition of Nef, if achieved, should interfere with the establishment and/or maintenance of latent reservoirs.

Despite the great promise, however, development of Nef inhibitors has been challenging (25). Two issues may have contributed to difficulties here. First, Nef lacks a defined pocket, and previous inhibitor development likely did not target the specific surface/pocket of Nef that is involved in Nef-mediated immune evasion. Second, previous efforts may have suffered from the conformational plasticity of Nef. As a master of protein–protein interactions, Nef is structurally versatile. Nef has a rigid core domain as well as two long flexible loops, one at the N-terminus and another close to the C-terminus. These flexible loops adopt different conformations when Nef interacts with different protein partners.

High-resolution structures solved by us have revealed the mechanistic details of Nef-mediated downregulation of MHC-I and CD4, respectively, which provided clues to overcome the above-mentioned challenges in Nef inhibitor development (26, 27). First, a conserved pocket of Nef was found to be involved in both activities of Nef, suggesting that this pocket of Nef should be the focus for inhibitor development (27). Second, these structures revealed the specific conformation of Nef involved in each downregulation and, importantly, how each conformation is achieved and/or stabilized by Nef’s association with the hijacked clathrin AP complex.

Nef downregulates MHC-I by intervening with the anterograde transport of newly synthesized MHC-I. At the *trans*-Golgi network, Nef orchestrates a three-molecule association between itself, the cytoplasmic domain of MHC-I (MHC-I_CD_), and the clathrin adaptor protein 1 (AP1) (26, 28, 29, 30, 31, 32). Through this interaction, the peptide-loaded MHC-I, instead of being transported to the cell surface, is redirected toward the endo-lysosomal pathway and eventually degraded in the lysosome. As revealed by our crystal structure, Nef binds exclusively to the C-terminal domain of the μ1 subunit (μ1^CTD^) of AP1. An elongated “furrow” is then formed at the Nef-μ1^CTD^ interface, and the MHC-I_CD_ binds snugly into it (Fig. 1*A*) (26). This MHC-I_CD_–binding site is of a distinctive shape and may be targeted by inhibitors to disrupt Nef-mediated MHC-I downregulation. MHC-I_CD_–binding covers an interface area of 1208 Å^2^, which is greater than what is believed to be optimal for inhibition by small molecules (binding surface ≤ 1000 Å^2^). However, given that the interaction between Nef, MHC-I, and μ1 is of a highly cooperative, delicate nature (26), we believe that the complex formation here should be sensitive to small molecule inhibitors. Inspired and guided by these structure-derived insights, we have designed, developed, and optimized a fluorescence polarization (FP) assay for identifying such inhibitors through high-throughput screening (HTS).

**Figure 1:**
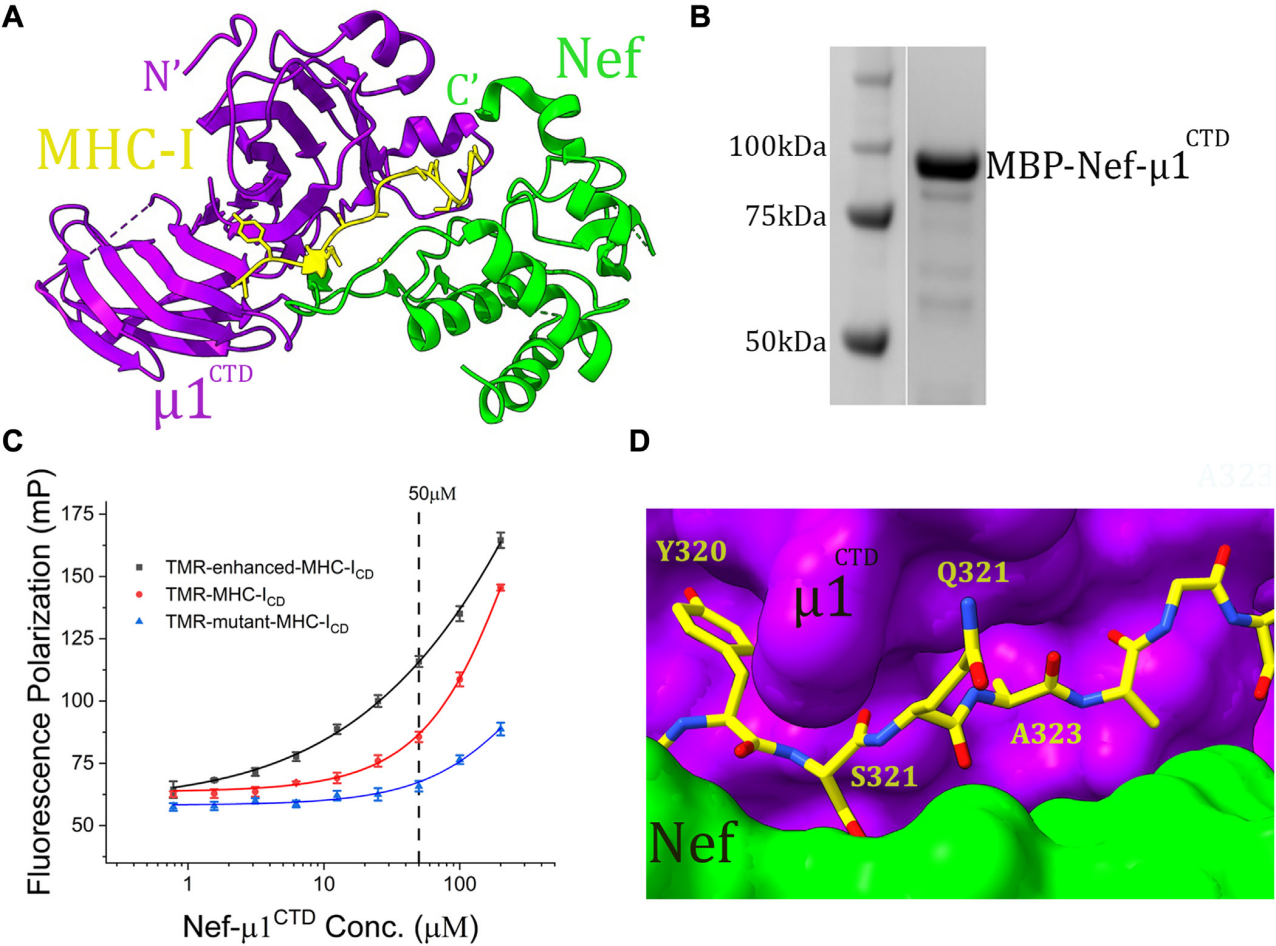
**Structure-guided design and initial tests of the FP assay.***A*, a cooperative three-protein binding places MCH-I_CD_ in a “furrow” formed at the interface between Nef and the C-terminal domain of the μ1 subunit of AP1 (PDB ID: 4en2). *B*, SDS PAGE analysis of purified MBP-Nef-μ1^CTD^. *C*, while Y320 of MHC-I binds to the tyrosine-binding pocket of μ1, the μ1 pocket designated for binding Φ is unoccupied because of the presence of a small Ala residue at position 323 of MHC-I. *D*, FP assays assessing the binding between the fluorescence probes and MBP-Nef-μ1^CTD^. Each data point is shown as the averaged FP from three independent samples ± SD. Each data series was fitted with a nonlinear regression curve using the one-site binding method. The TMR-mutant-MHC-I_CD_ curve is informative in two ways: at lower concentrations of MBP-Nef-μ1^CTD^, it indicates the baseline of the FP signal; at higher concentrations of MBP-Nef-μ1^CTD^, it shows the noise signal caused presumably by the light scattering of MBP-Nef-μ1^CTD^. FP signals generated using TMR-MHC-I_CD_ as the probe are above the baseline but modestly so. FP signals generated using TMR-enhanced-MHC-I_CD_ as the probe are sufficiently higher than the baseline.

## Results

### Construct design and initial FP assay using TMR-MHC-I_CD_

We reasoned that an FP assay using a fluorescently labeled MHC-I_CD_, if developed successfully, should work competently in HTS to identify the desired inhibitors—compounds that can associate into the pocket at the Nef-μ1^CTD^ interface and thus block the recruitment of MHC-I. We then followed the three-step protocol described by Moerke to develop such an FP assay (33). We first designed and commercially synthesized the fluorescent probe: a linear MHC-I_CD_ peptide with a fluorescent tag, tetramethylrhodamine (TMR), attached at the N-terminus. We then designed and created a Nef-μ1^CTD^ fusion construct capable of binding MHC-I_CD_ and thus suitable for the FP assay. As described above, the tri-molecular association between Nef, MHC-I_CD_, and μ1^CTD^ is cooperative in nature; in the absence of MHC-I_CD_, the binary association between Nef and μ1^CTD^ is of low affinity (K_D_ estimated to be in the micromolar range) and dissociation-prone (26). To ensure the formation of the binary complex between Nef and μ1^CTD^ and thus that of the MHC-I–binding pocket, we fused Nef to the N-terminus of μ1^CTD^ *via* a 20-amino-acid linker, which, according to the structure, should be flexible enough to not interfere with Nef’s binding with μ1^CTD^. The Nef-μ1^CTD^ fusion, carrying an N-terminal MBP tag, was expressed and purified to homogeneity in high yield (Fig. 1*B*).

For the FP assay, a working concentration of 50 nM was used for the TMR-MHC-I_CD_ probe because the emitted fluorescence intensity at this concentration is more than 10 times greater than the background signal (buffer only). The assay volume was miniaturized and kept at 15 μl for compatibility with HTS. As expected, when the concentration of the Nef-μ1^CTD^ fusion increased, the FP signal increased accordingly, which is consistent with the TMR-MHC-I_CD_ probe binding to the large Nef-μ1^CTD^ fusion protein leading to slower tumbling of the fluorophore and thus increased FP signal (Fig. 1*C*, red curve). In contrast, in the control experiment using a TMR-mutant-MHC-I_CD_ probe, which carries both Y320D and D327R mutations and is thus incapable of binding to Nef-μ1^CTD^, the FP signal stayed mostly at the background level, although it increased modestly after the Nef-μ1^CTD^ concentration increased beyond 50 μM (Fig. 1*C*, blue curve). Here, the above-background FP signal observed at concentrations of Nef-μ1^CTD^ higher than 50 μM indicates that a significant amount of noise exists in this concentration range. We believe that the noise signal is a result of light scattering caused by high concentrations of the large MBP-Nef-μ1^CTD^ fusion protein (Fig. 1*C*).

### Using TMR-enhanced-MHC-I_CD_ improves the competency of the FP assay

Although noise-free FP signal was successfully observed at low concentrations of Nef-μ1^CTD^ in our initial tests (Fig. 1*C*, red curve), the signal window (difference between the red and the blue curves, Fig. 1*C*) was very modest. We reasoned that the low affinity binding of TMR-MHC-I_CD_ to the Nef-μ1^CTD^ fusion could be what is limiting the signal window. We therefore sought to design a new probe that could bind the Nef-μ1^CTD^ fusion with higher affinity.

MHC-I contains a defective tyrosine-based sorting motif in its cytoplasmic tail. The canonical motif, denoted as YxxΦ, binds to the μ subunits of different clathrin AP complexes. It contains, in addition to Tyr, a large, hydrophobic residue (Φ: Leu, Ile, or Met). MHC-I_CD_, however, lacks such a hydrophobic residue at this position and has a small Ala residue here instead. Thus, while the Tyr residue within MHC-I_CD_, Y320, binds to the canonical Tyr-binding pocket of μ1, the μ1 pocket designated for binding Φ is left unfilled because of the presence of an Ala residue at position 323 of MHC-I (Fig. 1*D*). Such a feature dictates that, in the absence of Nef, MHC-I does not bind to μ1 efficiently and is therefore not trafficked through the AP1-dependent clathrin membrane trafficking pathway. This, however, offered an opportunity for our design of a new probe: converting the YxxA sequence within TMR-MHC-I_CD_ into the canonical YxxΦ motif should help improve the binding affinity between the fluorescent probe and the Nef-μ1^CTD^ fusion. We therefore created such a probe—replacing Ala323 with a Leu residue—and named it herein as TMR-enhanced-MHC-I_CD_. Gratifyingly, the use of TMR-enhanced-MHC-I_CD_ in the FP assay indeed resulted in higher-affinity binding: the K_D_ improved from 44.1 μM to 18.8 μM when the probe switched from TMR-MHC-I_CD_ to TMR-enhanced-MHC-I_CD_ (Fig. 1*C*). More importantly, the TMR-enhanced-MHC-I_CD_ probe helped improve the signal window of the assay in the region of low Nef-μ1^CTD^ concentration (Fig. 1*C*). The Nef-μ1^CTD^ concentration of 30 μM was selected, which gave a signal window of more than 50 mP and should allow sufficient sensitivity to competition (here, the equilibrium concentrations of the probe–protein complex and the free probe are calculated to be 30.7 nM and 19.3 nM, respectively).

### Assessment of the assay’s tolerance toward DMSO

**Figure 2:**
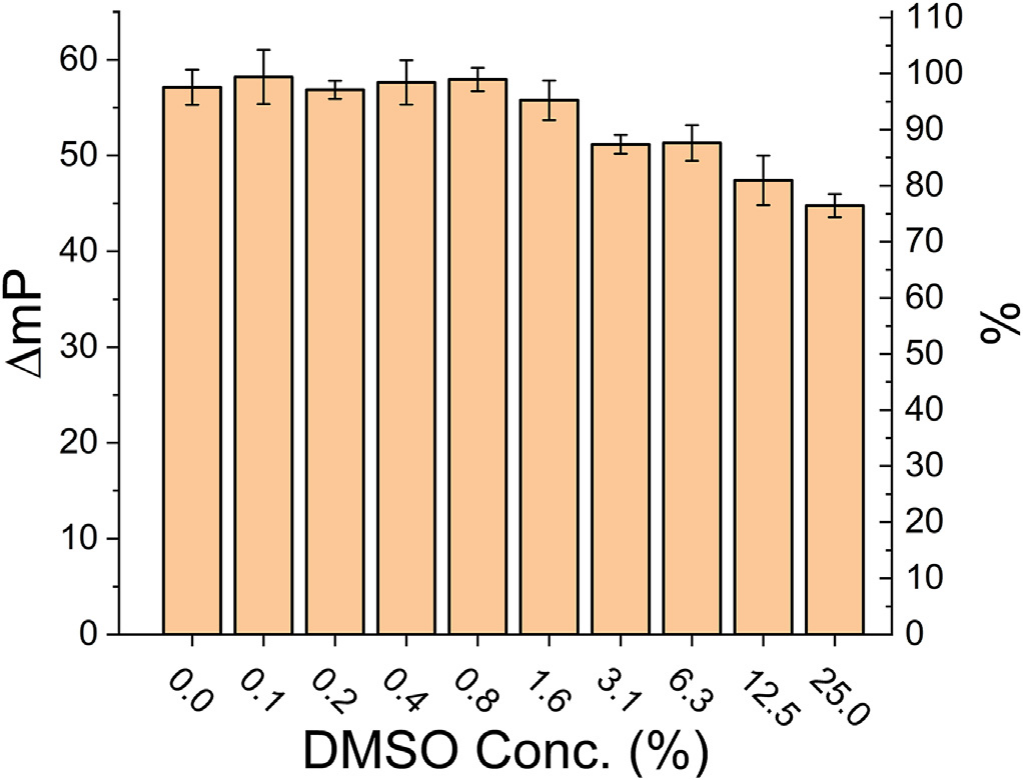
**Effect of DMSO on the FP assay.***Left* y axis: FP signal (mP) of the assay normalized by substracting out the background (buffer only) FP signal; the obtained ΔmP values here should correlate directly with binding. *Right* y axis: percentage of the FP signal correlated with binding (100% correlates with the FP signal of the optimized assay without DMSO added; 0% correlates with the background FP signal). Each data point is shown as the averaged FP of three independent samples ± SD. Analysis using one-way ANOVA indicates that this data is statistically significant (*p* < 0.0001). DMSO, dimethylsulfoxide.

Since compounds in most small molecule libraries are dissolved in dimethylsulfoxide (DMSO), we tested our assay for its tolerance of DMSO. As shown in Figure 2, DMSO concentrations of 1.6% or lower led to minimal, if at all, decrease of the FP signal. At DMSO concentrations of 3.1% or 6.3%, the FP signal window decreased by ∼10% only. Overall, our FP assay can be considered as stable at up to 6.3% of DMSO. Notably, for library screening, the assay needs to be tolerant of 1% of DMSO.

### The optimized assay is responsive to competitive binding of an inhibitor

**Figure 3:**
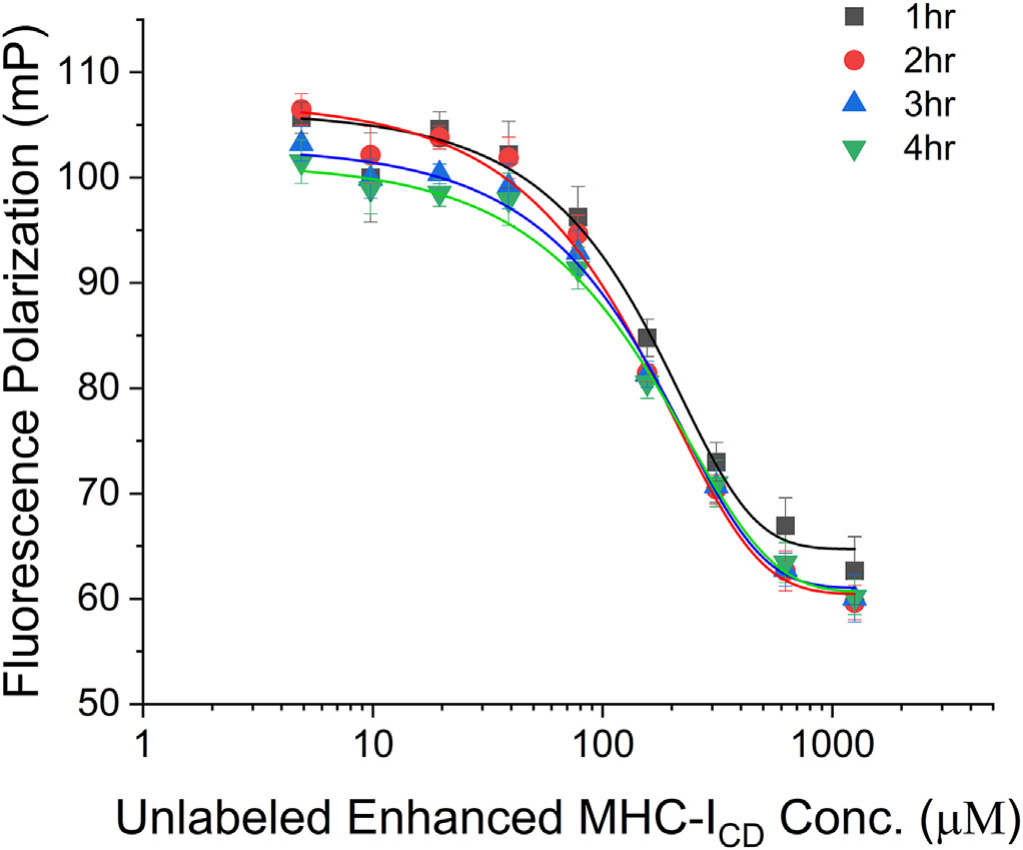
**Dose-dependent competition of the fluorescence-labeled probe by an unlabeled, MHC-I**_**CD**_**-mimetic peptide.** Competition was successfully observed after 1-h incubation, and the signals remained stable after 4 h. No further time points were shown. Each data point is shown as the averaged FP of three independent samples ± SD. Analysis using one-way ANOVA indicates that the data is statistically significant (*p* < 0.0001).

We next investigated whether our assay is sensitive to competitive inhibition. Here, an unlabeled, modified MHC-I_CD_ peptide was used as the competitor. The sequence of this competitor peptide is different from the authentic sequence of MHC-I_CD_ in the following ways. First, the same A323L mutation was introduced so that the complete YxxΦ motif was installed, which should allow this unlabeled peptide to compete efficiently with the TMR-enhanced-MHC-I_CD_ probe for binding. Second, to improve the solubility of the competitor peptide, we introduced three additional mutations in the MHC-I_CD_ sequence: Q322E, G325S, and S326D. According to the previous structure, these mutations should not interfere with Nef-μ1^CTD^ binding but should increase the peptide’s hydrophilicity. Adding this unlabeled, MHC-I_CD_-mimetic peptide as the competitor indeed led to a dose-dependent decrease of the FP signal, consistent with the expected displacement of the TMR-enhanced-MHC-I_CD_ probe by the competitor (Fig. 3).

### The FP assay is robust and suitable for HTS

**Figure 4:**
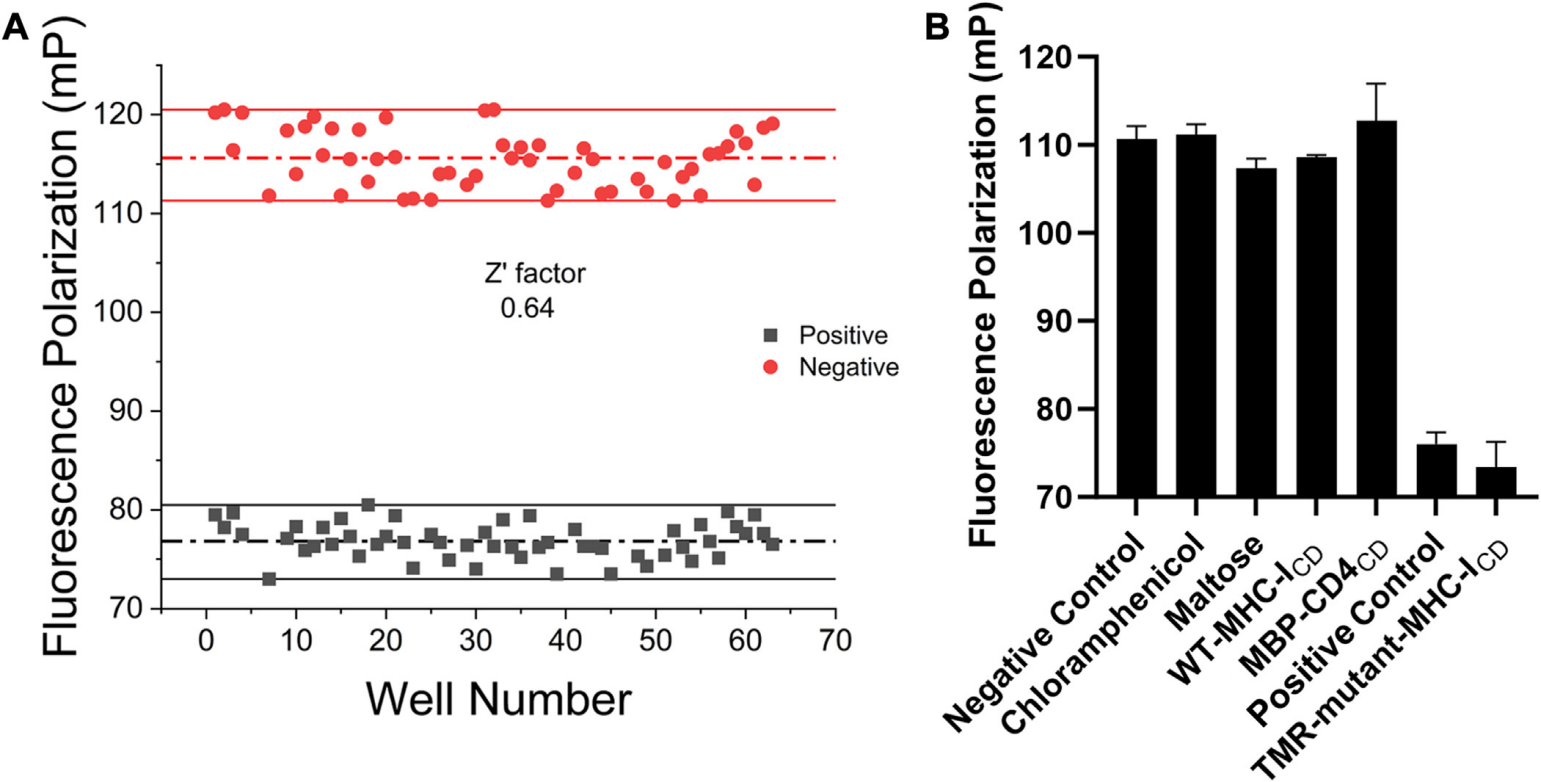
**Assessing the assay’s suitability for HTS.***A*, measurement of the Z′ factor. The mean values of positive and negative controls are indicated by the *dashedlines*, while *solid* lines indicate the range where data points were considered. *B*, tool compounds did not cause noise in the assay. Chloramphenicol, maltose, WT-MHC-I_CD_ peptide (319–330 of MHC-I), and MBP-CD4_CD_ (394–419) was each added to the assay solution to a final concentration of 300 μΜ (final dimethylsulfoxide concentration = 3%). Negative and positive controls were prepared the same way as in A.

To assess whether our assay is competent for HTS, we calculated the *Z*′ factor. For the positive control, 600 μM of the unlabeled, modified MHC-I_CD_ peptide was used to ensure the complete displacement of the TMR-enhanced-MHC-I_CD_ probe. From 60 positive controls and 60 negative controls (Nef-μ1^CTD^ with TMR-enhanced-MHC-I_CD_), the *Z*′ factor was calculated to be 0.64, indicative of an excellent assay for HTS (Fig. 4*A*).

To further assess the assay’s specificity and suitability for screening, we tested the following tool compounds/molecules in the assay: chloramphenicol (a secondary metabolite), maltose (a carbohydrate), WT-MHC-I_CD_ (a peptide), and MBP-CD4_CD_ (a chimeric protein containing MBP and the cytoplasmic tail of CD4). MBP-CD4_CD_ should help validate the specificity of our assay. As mentioned earlier, although Nef downregulates CD4, the CD4-binding pocket should be properly formed only when Nef is in complex with clathrin AP2. In contrast, the MHC-I–binding pocket formed at the Nef-μ1 interface is of a distinct shape, which should not allow CD4 binding. The WT-MHC-I_CD_ peptide is less capable than the TMR-enhanced-MHC-I_CD_ probe in binding the MBP-Nef-μ1^CTD^ fusion; thus, addition of WT-MHC-I_CD_ should not decrease the FP signal in any way efficient or at all. Furthermore, maltose binds into the active site of MBP but should not bind the Nef-μ1^CTD^ portion of the fusion protein; this compound can therefore test how our assay would respond to binding occurring at a site other than the targeted MHC-I_CD_–binding pocket. As expected, addition of these molecules at 300 μΜ did not lead to any meaningful decrease of the FP signal (Fig. 4*B*). Importantly, the assay also did not suffer from any significant noise (Fig. 4*B*). Furthermore, the background of the assay showed no fluctuation at all when these molecules were added (not shown). Results here were highly reproducible when the experiment was performed on different plates and different days. Overall, the measured *Z′* factor and the tests with tool compounds/molecules together indicate that our assay is competent and suitable for HTS (33).

## Discussion

We have developed a robust, HTS-compatible FP-based assay for screening small molecule inhibitors that directly target the MHC-I–binding pocket of the Nef–AP1 complex. Our work here was enabled and facilitated by the crystal structure reported by us previously (26). The highly cooperative nature of the binding, as revealed by the structure, prompted us to design a fusion protein to favor the formation of the MHC-I-binding “furrow” at the Nef-μ1 interface (Fig. 1AB). In addition, the structural revelation that MHC-I binds to the conserved pocket on μ1 using a “defective” YxxΦ motif inspired us to “restore” a canonical sorting motif in our MHC-I_CD_–based fluorescent probe, which was key in enabling our assay to achieve the desired signal window (Fig. 1CD). Our work here further illustrates how high-resolution structures may inspire efforts toward developing drugs against challenging targets.

There are, however, some limitations with our assay here, which should be taken into account during the operation of the HTS. First, in the competition experiment, the FP signal was stable up to 4 h after addition of the competitor (Fig. 3); longer incubation led to a gradual decrease of the FP signal, although the dose-dependent trend was maintained (not shown). This time window is large enough for assay setup and plate reading but is nonetheless a limiting factor. To accommodate this, we may need to limit the number of libraries to be screened on a single day of operation. Second, according to our data (Fig. 3), high concentrations of the competitor were needed to displace the TMR-enhanced-MHC-I_CD_ probe (IC_50_ = ∼170 μM). We suspect that the effective concentration of our competitor could be much lower than the apparent concentration. We have noticed during our experiments that the competitor—unlabeled enhanced MHC-I_CD_ peptide—sometimes forms precipitates, indicating that it still has solubility issues. It is therefore possible that only a portion of this peptide was in the aggregation-free state and thus capable of competing with the TMR-enhanced-MHC-I_CD_ probe for binding Nef-μ1^CTD^. If this is true, then the actual IC_50_ would be smaller and may thus look more reasonable. Whether or not the measured IC_50_ of unlabeled enhanced MHC-I_CD_ peptide is an understatement of its true competency as a competitor, it remains safe to conclude that this optimized assay is capable of detecting competitive binding at the targeted pocket. However, if the measured IC_50_ is indeed high and over 100 μM competitor is needed to induce a statistically significant drop of the FP signal, it could mean that low-affinity binders may be missed as hits during HTS using this assay.

Parallel to this work, we have also developed, as reported in the companion publication (34), an FP assay for screening small molecule inhibitors that could disrupt HIV-1 Nef-mediated CD4 downregulation. We will use the two assays in parallel in HTS, which should help us identify true inhibitors of Nef with unique specificities (active against one or both functions of Nef).

## Experimental procedures

### Fusion protein design, expression, and purification

The Nef-μ1^CTD^ fusion was constructed by fusing HIV-1 Nef (59–206, NL4.3) to the C terminal domain of μ1 subunit of AP1 *via* a flexible linker of 20 amino acids. Gene encoding the above Nef-μ1^CTD^ fusion was cloned into a modified pMAT9 expression vector with a 6xHis tag introduced at the C-terminus of the fusion protein. *Escherichia coli* cells transformed with the plasmid were grown at 37 °C till A_600_ reached 0.8. Protein expression was then induced with 0.1 mM IPTG and continued at 16 °C overnight. Cells were then lysed using sonication. The protein of interest was purified sequentially through a Ni-NTA affinity column, a MBP affinity column, a HiTrap Q anion exchange column, and finally a Superdex 200 size exclusion column.

### Fluorescence polarization assay using Nef-μ1^CTD^ and the TMR-enhanced-MHC-I_CD_ peptide

Purified MBP-Nef-μ1^CTD^ was buffer exchanged into the assay buffer (50 mM Tris, 150 mM NaCl, 0.5 mM DTT, 0.01% Triton X-100, pH 8.0). A stock protein solution of 120 μM Nef-μ1^CTD^ was then prepared and was subsequently used to create different dilutions. Assays were carried out in *Corning* 384-well black microplates (3820). In each well, 50 nM TMR-labeled MHC-I_CD_ peptide was mixed with MBP-Nef-μ1^CTD^ at varied concentrations in a total volume of 15 μl. The plate was incubated for 1∼2 h at room temperature with minimal exposure to light. FP was then measured using the *EnVision* plate reader (*PerkinElmer*) with excitation at 535 nm and emission at 595 nm. Experiments were done in triplicates. Data was fitted to nonlinear regression and plotted as a function of protein concentration in a logarithmic scale using *OriginLab*.

The probes used were synthesized by *GenScript*, including the following: TMR-MHC-I_CD_: TMR-SYSQAAGSDSAQ; TMR-enhanced-MHC-I_CD_: TMR-SYSQLAGSDSAQ; TMR-mutant-MHC-I_CD_: TMR-SDSQAAGSRSAQ. These probes are stable at room temperature on the bench for at least 20 h.

For calculating the dissociation constants (K_D_), the noise, represented by the FP signals generated with TMR-mutant-MHC-I_CD_ being used as the probe, was first subtracted from the measured FP signals with TMR-MHC-I_CD_ and TMR-enhanced-MHC-I_CD_, respectively. The adjusted data was then fitted to nonlinear regression for one-site binding using the following equation: Y = B_max_∗X/(K_D_ + X) (B_max_: maximum specific binding in the same units as Y).

### DMSO tolerance

Assay solutions were prepared containing 30 μM MBP-Nef-μ1^CTD^, 50 nM TMR-enhanced-MHC-I_CD_, and different concentrations of DMSO (0–25%). After incubation at room temperature for 2 h, FP values were measured and recorded. Experiments were done in triplicates.

### Competition of TMR-enhanced-MHC-I by unlabeled enhanced-MHC-I

For competition using unlabeled enhanced-MHC-I_CD_ peptide, a stock solution of 1.2 mM unlabeled enhanced-MHC-I_CD_ peptide was first prepared. The stock solution was then serial-diluted (2-fold each) 10 times. For making the final assay solutions, MBP-Nef-μ1^CTD^ and TMR-enhanced-MHC-I_CD_ were first mixed and incubated at room temperature for 1 h. Then, unlabeled enhanced-MHC-I_CD_ peptide was added (final concentrations: 30 μM MBP-Nef-μ1^CTD^, 50 nM of TMR-enhanced-MHC-I_CD_, and varied concentrations of unlabeled enhanced-MHC-I_CD_ peptide). The plate was incubated at room temperature and read at different time points. FP values were recorded. All experiments were done in triplicates.

### Determination of the *Z′* factor

Negative control contains 30 μM of MBP-Nef-μ1^CTD^, 50 nM TMR-enhanced-MHC-I_CD_, and 2% DMSO. Positive control contains 30 μM MBP-Nef-μ1^CTD^, 50 nM TMR-enhanced-CD4_CD_, ∼600 μM unlabeled enhanced-MHC-I_CD_, and 2% DMSO. Samples of positive controls and negative controls (60 wells each) were prepared in a 384-well plate and incubated at room temperature for 2 h. FP values were recorded using the plate reader. The *Z′* factor was calculated using the following equation:

$$Z^{\prime }=1\;-\;\frac{3\;\times \;\left(SD_{N}\;+\;SD_{P}\right)}{\left|\mu_{N}\;-\;\mu_{P}\right|}$$

Where $\mu_{N}$ and $\mu_{P}$ are the averages of mP values of negative and positive controls, respectively. $SD_{N}$ and $SD_{P}$ are the SDs.

### Data analysis

Nonlinear regression fitting of the FP data was done using *OriginLab*. Statistical analysis was performed using Ordinary one-way ANOVA in *GraphPad Prism*. A *p* value of < 0.05 is considered statistically significant.

## Data availability

All data generated or analyzed during this study are included in this published article.

## Conflict of interest

The authors declare that they have no conflicts of interest with the contents of this article.
